# Number of Medical Graduates and Licentiates Dnring the Last Year

**Published:** 1845-08-01

**Authors:** 


*Number of Medical Graduates and Licentiates dnring the last year.*—The number who have received Diplomas from the Medical Colleges, respectively, of this State, during the last year, are as follows:

| | |
|---|---|
| College of Physicians and Surgeons, | 37 |
| University of New-York, | 92 |
| Albany Medical College, | 16 |

These we find among the transactions of the State Medical Society, furnished agreeably to a resolution requesting annual reports. The Geneva College, which did not report, graduated, as we learn from Prof. Hamilton, 46. The whole number is 191.—Seventeen county societies, reported only 8 licentiates.

